# Sociodemographic and health-related determinants of influenza vaccine nonreceipt among US adults: A cross-sectional analysis of the 2022 National Health Interview Survey

**DOI:** 10.1097/MD.0000000000041854

**Published:** 2025-03-14

**Authors:** Ruemon Bhattacharyya, Samuel J. Katz, Mehul Bhattacharyya, Anna L. Miller, Larry E. Miller

**Affiliations:** a University of California Los Angeles. Los Angeles, CA; b Arizona State University, Tempe, AZ; c Department of Biostatistics, Miller Scientific, Johnson City, TN.

**Keywords:** flu, influenza, NHIS, vaccination, vaccine

## Abstract

The purpose of this study was to determine the prevalence and factors associated with influenza vaccine nonreceipt among adults in the United States. This nationally representative, cross-sectional study analyzed self-reported data on influenza vaccination status and sociodemographic, physical health, and health care access factors among 27,189 adults from the 2022 National Health Interview Survey. The primary outcome was influenza vaccination nonreceipt in the past 12 months. A multivariable logistic regression model evaluated associations between 19 sociodemographic, physical health, and health care access variables with influenza vaccine nonreceipt. Shapley Additive Explanations assessed the relative importance of covariates associated with nonreceipt in the regression model. The population-weighted analysis indicated 52.8% influenza vaccination nonreceipt among US adults. The logistic regression model identified 15 covariates as significantly associated with vaccine nonreceipt. When evaluating the relative importance of the variables, the key determinants of vaccine nonreceipt were younger age, lack of doctor visits in the past year, and lower educational attainment. Influenza vaccine nonreceipt was 92.4% among adults with all 3 characteristics compared to only 16.0% among those with none of the characteristics. In conclusion, influenza vaccine coverage in the overall US adult population remains well below public health goals. Significant disparities persist among subgroups like younger adults, those without recent health care visits, and individuals with lower educational attainment. There is an urgent need for expanded outreach efforts targeting vulnerable populations to address disparities in vaccine uptake.

## 1. Introduction

Seasonal influenza continues to cause substantial morbidity and mortality in the United States despite long-standing recommendations for annual influenza vaccination among persons aged ≥ 6 months without contraindications.^[1]^ In the United States, the overall burden of influenza for the 2021 to 2022 season included 11 million flu illnesses, 5.1 million flu-related medical visits, 120,000 flu-related hospitalizations, and 6300 flu deaths.^[2]^ Vaccination protects against influenza and associated complications for individuals and communities. Recent estimates indicate vaccinations prevented 7.5 million influenza cases, 105,000 influenza-associated hospitalizations, and 6300 influenza-associated deaths in the United States.^[3]^ However, national influenza vaccine coverage has plateaued well below public health goals over the past decade.^[4]^ Improving population-level acceptance and equitable access to influenza vaccination is critical for more robust and sustained community protection against influenza.

As part of the Healthy People 2030 national public health initiative to improve the well-being of Americans over the next decade, targets have been established to increase annual influenza vaccine coverage to 70% among adults.^[4]^ However, recent population-based surveys demonstrate coverage levels falling substantially below this goal, with annual vaccination coverage in adults ranging from 37% to 50% between 2010 and 2022.^[3]^ Examining disparities in vaccine uptake across sociodemographic groups may help identify populations facing disproportionate risks of influenza due to lower vaccination rates. This, in turn, may help to raise awareness of coverage inequities while also identifying interventions and policies to promote health equity. While prior studies have examined associations between select factors with influenza vaccination rates,^[5,6]^ a comprehensive analysis of sociodemographic, physical health, and health care access determinants may further help to identify key determinants of influenza vaccination patterns. This study utilized the most recent self-reported influenza vaccination data among adults from the National Health Interview Survey (NHIS), an annual nationwide US survey on health care access and health behaviors, to determine the prevalence of influenza vaccine nonreceipt among US adults. By examining multiple sociodemographic and health-related determinants in this nationally representative US sample, we also aimed to identify key factors associated with lower influenza vaccine coverage.

## 2. Methods

### 2.1. Study design and participants

This cross-sectional observational study analyzed data from the 2022 NHIS. The NHIS is an annual household interview survey providing health information on the noninstitutionalized civilian population in the US. The NHIS randomly sampled US households in all 50 states and the District of Columbia using a multistage cluster sampling design with geographic stratification. One adult (>18 years) was randomly selected from each sampled household to participate. Excluded populations were active-duty military personnel, civilians living on military bases, individuals in long-term care or correctional facilities, and persons without a fixed household address. Interviews for the 2022 NHIS were conducted from January 1 to December 31, 2022. In total, 27,651 adults completed interviews, representing a response rate of 47.7%. All participants provided informed consent under protocols approved by the National Centers for Health Statistics Ethics Review Committee. This study followed the Strengthening the Reporting of Observational Studies in Epidemiology reporting guideline^[7]^ and the Preferred Reporting Items for Complex Sample Survey Analysis.^[8]^

### 2.2. Outcomes

The primary outcome of this study was self-reported nonreceipt of influenza vaccination in the past 12 months. Of note, in the US, routine annual influenza vaccination is recommended for all persons aged 6 months or older who do not have contraindications.^[9]^ Covariates were selected based on known associations with influenza vaccine coverage.^[5,6]^ Sociodemographic characteristics included geographic indicators of US region and urban-rural classifications,^[10]^ age, sex, race/ethnicity (Asian, Black, White, Hispanic, American Indian/Alaska Native, Other/multirace), marital status, and income-to-poverty ratio as a percentage of federal poverty guidelines. Physical health factors included self-reported overall health status and comorbidities including diabetes, cancer, lung disease, coronary heart disease, and disability status derived from the Washington Group Short Set Composite Disability Indicator.^[11]^ Health care access variables included health insurance coverage, having a usual place for health care, time since the last doctor visit, and whether needed medical care or prescription medications were declined over the past year due to cost.

### 2.3. Statistical analysis

A complex sample analysis incorporated sampling weights, strata, and cluster variables to generate nationally representative estimates from the survey data. Sampling weights were calculated as the inverse probability of selection, with adjustments to account for survey nonresponse. Linearized variance estimates derived through Taylor series expansion were used to account for the multistage stratified cluster sampling design. Multivariable logistic regression models examined associations of sociodemographic, physical health, and health care access variables with influenza vaccine nonreceipt, and were reported as odds ratios and 95% confidence intervals. Because identifying trivial statistically significant associations is common in population-based studies, Shapley Additive Explanations (SHAP) were additionally utilized to determine the relative importance of each variable when accounting for all possible interactions. This machine-learning technique decomposes the model output by calculating SHAP values derived from all possible variable subsets. The SHAP value indicates the explanatory power of each variable on a 0 to 100% scale.^[12]^ A SHAP value threshold of 10% was set to identify independently influential variables since this cutoff represents twice the hypothetical average influence of 5% that would be expected if all variables exhibited equal predictive importance. Variables achieving statistical significance (*P* < .05) and with sufficient explanatory contribution (SHAP > 10%) were considered important determinants.^[13]^ These factors were subsequently evaluated in a subgroup analysis. Statistical analyses were performed by a statistician author using Stata, v18 (StataCorp).

## 3. Results

### 3.1. Participant characteristics

Influenza vaccination status was self-reported for 27,189 (98.3%) participants. The mean participant age was 53 ± 18 years, and 54% were female. In the unweighted descriptive analysis, 48.3% reported being unvaccinated in the prior year. Vaccination nonreceipt was higher in the South and nonmetropolitan areas, as well as among younger adults, males, Hispanic or Black individuals, those with lower educational attainment, unmarried individuals, and those with lower household income (all *P* < .001; Table 1). All health and health care access characteristics were also statistically associated with vaccination nonreceipt (all *P* < .001). Individuals reporting better overall health status and an absence of comorbidities and disability were less likely to be vaccinated than those with poorer health profiles. Additional factors linked to vaccine nonreceipt were lacking health insurance, not having a usual place for health care, not seeing a doctor within the past year, and skipping needed medical care and medications in the past year due to cost (Table 2).

**Table 1:** Sociodemographic characteristics of 27,189 adults in the 2022 National Health Interview Survey by influenza vaccine nonreceipt*.

Variables	Unvaccinated (%)†	Proportion of subgroup unvaccinated (%)
Overall sample	48.3	100
Geographic characteristics, %		
Household region		
Northeast	41.7	14.3
Midwest	45.9	20.7
West	48.4	24.9
South	52.7	40.0
Urban-rural county classification		
Large central metro	47.8	29.7
Large fringe metro	46.6	22.6
Medium and small metro	47.6	30.5
Nonmetropolitan	53.3	17.2
Sociodemographic characteristics, %		
Age, yr		
18–34	65.2	28.0
35–64	53.9	52.7
≥65	29.2	19.4
Sex		
Female	44.8	50.5
Male	52.5	49.5
Race		
Asian	43.0	5.3
White	44.3	60.7
AIAN	49.5	0.7
Other/multirace	57.5	2.2
Black	58.5	13.4
Hispanic	60.0	17.7
Education level		
Less than high school	58.1	13.0
High school	53.4	56.6
Bachelor’s degree	43.4	20.7
Master’s degree	33.5	7.7
Professional degree	24.6	2.0
Marital status		
Married	42.8	41.3
Unmarried	52.7	58.7
Income		
<200% FPG	56.6	32.1
200–399% FPG	51.5	30.8
≥400% FPG	41.0	37.1

AIAN=American Indian and Alaskan Native, FPG=federal poverty guidelines.

* Values are unweighted descriptive statistics from the study sample.

† Statistically significant (*P* <.001) differences in influenza vaccine nonreceipt identified among all subgroups.

**Table 2:** Health characteristics and health care access among 27,189 adults in the 2022 National Health Interview Survey by influenza vaccine nonreceipt*.

Variables	Unvaccinated (%)†	Proportion of subgroup unvaccinated (%)
Health characteristics		
General health status		
Excellent	53.1	22.5
Very good	47.5	33.8
Good	48.0	29.7
Fair	45.1	10.8
Poor	42.1	3.2
Diabetes		
Yes	33.4	7.4
No	50.1	92.6
Cancer		
Yes	29.7	7.7
No	51.0	92.3
Lung disease		
Yes	38.8	4.4
No	48.9	95.6
Coronary heart disease		
Yes	29.0	3.8
No	49.6	96.2
Disabled		
Yes	41.8	8.9
No	49.1	91.1
Healthcare access		
Health insurance coverage		
Yes	45.5	87.2
No	82.0	12.8
Have a usual place for health care		
Yes	45.5	85.5
No	76.5	14.5
Time since last saw a doctor		
< 1 year	43.7	77.3
1–2 years	70.4	10.7
2–3 years	73.3	4.3
3–5 years	83.1	3.6
5 + years, or never	86.2	4.2
Declined needed medical care due to cost		
No	47.3	92.2
Yes	65.5	7.8
Declined needed prescription medication due to cost		
No	48.0	94.1
Yes	53.7	5.9

* Values are unweighted descriptive statistics from the study sample.

† Statistically significant (*P* < .001) differences in influenza vaccine nonreceipt identified among all subgroups.

### 3.2. Population-weighted influenza vaccine nonreceipt

**Figure 1.**
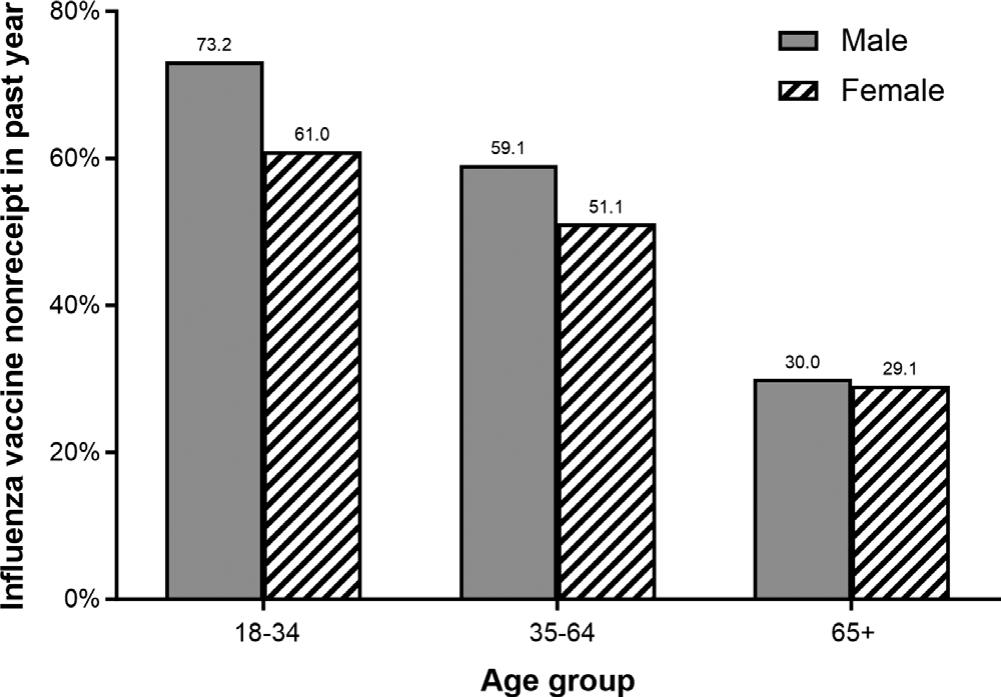
Influenza vaccine nonreceipt among adults in the 2022 National Health Interview Survey by sex and age group.

In the population-weighted analysis, the overall prevalence of influenza vaccine nonreceipt among US adults was 52.8%, which varied by age and sex (Fig. 1).

### 3.3. Factors associated with influenza vaccine nonreceipt

**Figure 2.**
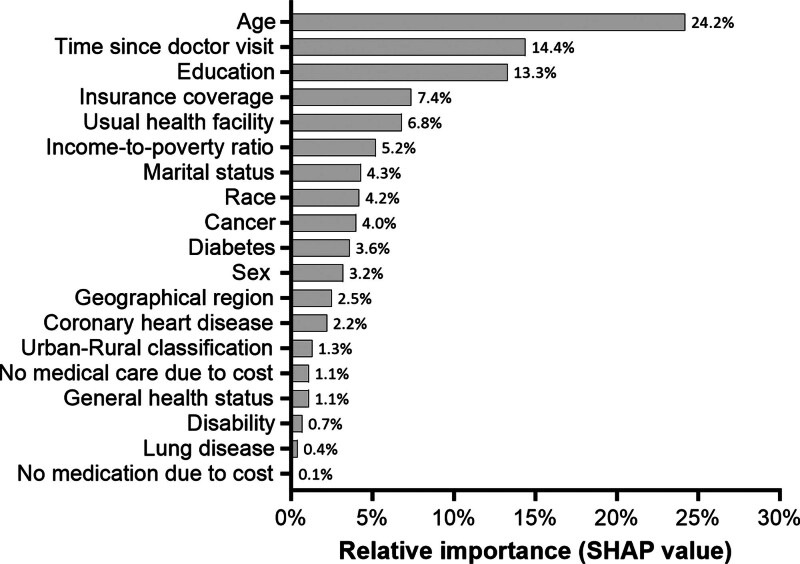
Relative importance of covariates in a multivariable logistic regression model to predict influenza vaccine nonreceipt among adults in the 2022 National Health Interview Survey. A SHAP value ≥ 10% identifies independently influential variables in the model. SHAP = Shapley Additive Explanations.

The multivariable logistic regression analysis revealed that 15 of the 19 examined covariates remained statistically associated with vaccination nonreceipt. Variables that were not associated with vaccination nonreceipt included general health status, lung disease, disability, and medication cost (Table 3). When additionally evaluating the relative explanatory importance of these variables in the model, only 3 characteristics met the dual *P* value and SHAP value criteria for predicting influenza vaccine nonreceipt: age, time since last doctor visit, and education (Fig. 2).

**Table 3:** Multivariable logistic regression of factors associated with influenza vaccine nonreceipt among adults in the 2022 National Health Interview Survey*.

Variables	Unit of measure	Odds ratio	95% CI	*P* value
Geographic characteristics				
Household region	Northeast	1 (Reference)	—	<.001
	Midwest	1.07	0.95–1.20	
	West	1.19	1.06–1.34	
	South	1.38	1.24–1.54	
Urban-rural county classification	Large central metro	1 (Reference)	—	<.001
	Medium and small metro	1.12	1.02–1.23	
	Large fringe metro	1.17	1.06–1.29	
	Nonmetropolitan	1.40	1.25–1.58	
Sociodemographic characteristics				
Age	≥65 years	1 (Reference)	—	<.001
	35–64	2.40	2.24–2.58	
	18–34	2.82	2.56–3.12	
Sex	Male vs. female	1.35	1.27–1.44	<.001
Race	Asian	1 (Reference)	—	<.001
	Hispanic	1.23	1.04–1.46	
	White	1.25	1.08–1.44	
	Other/multirace	1.26	0.95–1.66	
	AIAN	0.76	0.48–1.21	
	Black	1.76	1.48–2.11	
Educational attainment	Professional degree	1 (Reference)	—	<.001
	Master’s degree	1.89	1.56–2.28	
	Bachelor’s degree	2.42	2.02–2.89	
	High school	3.64	3.03–4.37	
	Less than high school	4.13	3.36–5.09	
Marital status	Unmarried vs. married	1.26	1.17–1.35	<.001
Income	≥400% FPG	1 (Reference)	—	<.001
	200–399% FPG	1.26	1.17–1.36	
	<200% FPG	1.34	1.22–1.47	
Health characteristics				
General health status	Poor	1 (Reference)	—	.06
	Fair	1.00	0.82–1.23	
	Good	1.06	0.86–1.31	
	Very good	1.08	0.88–1.34	
	Excellent	1.21	0.97–1.50	
Diabetes	No vs yes	1.56	1.40–1.74	<.001
Cancer	No vs yes	1.33	1.20–1.47	<.001
Lung disease	No vs yes	1.15	0.99–1.33	.07
Coronary heart disease	No vs yes	1.38	1.19–1.59	<.001
Disabled	No vs yes	1.12	0.98–1.27	.09
Healthcare access characteristics				
Health insurance coverage	No vs yes	1.78	1.54–2.06	<.001
Have a usual place for health care	No vs yes	1.66	1.45–1.90	<.001
Time since last doctor visit	<1 year	1 (Reference)	—	<.001
	1–2 years	1.94	1.70–2.20	
	2–3 years	2.05	1.66–2.53	
	3–5 years	3.24	2.49–4.23	
	5 + years, or never	2.93	2.10–4.09	
Declined needed medical care due to cost	Yes vs no	1.23	1.05–1.45	.01
Declined needed prescription medication due to cost	No vs yes	1.02	0.88–1.18	.80

Values are weighted and nationally representative of the US adult population.

AIAN = American Indian and Alaskan Native, CI = confidence interval, FPG = federal poverty guidelines.

### 3.4. Subgroup analysis

The subgroups at highest risk for vaccine nonreceipt were younger adults, those with a longer time since a doctor visit, and those with lower educational attainment. Adults exhibiting all 3 characteristics had a 92.4% prevalence of vaccine nonreceipt. In contrast, among older adults with higher education attainment who saw a doctor within the last year, influenza vaccine nonreceipt was only 16.0% (Table 4).

**Table 4:** Influenza vaccine nonreceipt across risk groups in the 2022 National Health Interview Survey*.

Age	Time since doctor visit	Educational attainment†		
		High	Medium	Low
≥65	<1 year	16.0	23.0	31.1
	1–3 years	40.3	48.1	62.1
	≥3 years	60.2	58.1	76.9
35–64	<1 year	34.2	44.9	56.8
	1–3 years	47.2	61.5	80.4
	≥3 years	69.8	77.9	87.2
18–34	<1 year	38.4	49.5	68.5
	1–3 years	42.0	69.4	83.6
	≥3 years	70.0	83.8	92.4

* Values are weighted percentages and nationally representative of the US adult population. The weighted population prevalence of influenza vaccine nonreceipt was 52.8%.

† High educational attainment includes Master’s, doctorate, or professional degree; medium includes Bachelor’s degree; low includes less than Bachelor’s degree.

## 4. Discussion

In this nationally representative 2022 study of US adults, the overall prevalence of influenza vaccine nonreceipt in the prior year was 52.8%, considerably above the Healthy People 2030 national target of 30% nonreceipt.^[4]^ This stagnation demonstrates failure to meaningful improve influenza vaccine coverage over the past decade, as influenza vaccine nonreceipt has ranged from 50% to 63% annually since 2010.^[3]^ Moreover, substantial disparities persist across sociodemographic subgroups, with lower vaccination coverage among younger adults, populations with lower health care utilization, and individuals with lower educational attainment. These findings suggest an urgent need to further investigate barriers underlying the persistent low influenza vaccine uptake both in the general population as well as among vulnerable groups. These actions could inform specific interventions to improve access and acceptance of routine annual influenza vaccination in the US population.

Younger adults exhibited significantly lower vaccine uptake, especially those with low educational attainment whose nonreceipt prevalence reached 92.4% for those with a longer time since their last doctor visit. While reasons for vaccine nonreceipt were not collected in this study, younger individuals may underestimate personal risk and fail to appreciate vaccination benefits due to perceived invulnerability, knowledge gaps, or health literacy limitations. Prior studies also demonstrate that willingness for vaccination improves with even modest financial incentives in this group,^[14]^ suggesting potential affordability and accessibility barriers. Individuals with extended periods between physician visits also showed higher vaccine nonreceipt. This finding suggests potential systemic obstacles in engaging certain individuals in preventive services, potentially reflecting attitudinal factors or financial/health care access limitations that discourage routine care and preventive health services utilization. Yet even among those with a recent provider visit, 44% still declined vaccination, indicating additional unmet needs regarding vaccine education, affordability, or access.

Lower educational attainment was linked with higher rates of vaccine nonreceipt, potentially reflecting broader health care access inequities. Individuals with less education tend to face resource gaps related to economic stability, social connections, health-promoting behaviors, and quality health care.^[15]^ Such gaps contribute to the education-based health disparities in the United States,^[15]^ and likely influenced the association between education and influenza vaccine uptake observed in this study. In support of this hypothesis, the logistic regression analysis revealed that other barriers like lower income, insurance gaps, and minority race had significant, yet modest, independent influence in the model. Education level may also proxy for health literacy, influencing vaccine decision-making and behaviors.^[16]^ Although not directly assessed in this study, individuals with less education typically have poorer health literacy, which is a known obstacle to shared decision-making.^[17]^ Providers have been shown to preferentially engage in shared decision-making with patients who seem more capable of participating in the process.^[18]^ Yet vulnerable groups with lower health literacy stand to benefit the most from informed discussions supporting personalized vaccination decisions. The Centers for Disease Control and Prevention and state health departments promote influenza vaccination through a reminder system that sends automated phone calls, text messages, emails, or postcards annually to registered patients through participating health care providers before and during influenza season. They also maintain a vaccine finder website, offer clinical provider recommendation toolkits, and distribute multilingual educational materials through health care settings and pharmacies. Despite these efforts, influenza vaccine uptake is largely unchanged in recent years. Overall, advancing equity in influenza vaccine coverage will require focused approaches that promote universal access to vaccine education while proactively reaching disadvantaged subgroups to understand and mitigate their specific concerns and barriers regarding immunization.

Although reasons for influenza vaccine nonreceipt were unreported in the 2022 NHIS survey, prior research has reported common barriers to influenza uptake including perceiving low-priority status, doubting benefits, concerns about adverse effects, disagreement with national recommendations, and inconvenience.^[19,20]^ Many adults incorrectly feel that guidelines only apply to high-risk groups. Prior to 2010, influenza vaccination was recommended for all healthy people excluding those aged 19 to 49 years. Since 2010, annual vaccination has been recommended for all adults without contraindications. Public health campaigns promoted this universal recommendation while also emphasizing the particular importance of vaccination for pregnant women, adults aged ≥ 65 years, health care workers, and those with chronic medical conditions.^[9]^ This reveals potential gaps in awareness of personal and community vaccine benefits as well as heightened concerns about potential harms. There appears to be a continued need to clearly convey universal adult eligibility recommendations, while emphasizing the safety profile of influenza vaccination. In addition, health care providers may indirectly perpetuate vaccine nonreceipt by failing to recommend vaccination to their patients.^[21]^ Since the vaccine uptake rate of physicians mimics that of the general population, physician perceptions likely influence patient decisions.^[22,23]^ Thus, health care providers should routinely assess vaccination status and unequivocally recommend the influenza vaccine for all adults without contraindications.

Ultimately, a multifaceted strategy integrating system-level reforms to address health care inequities and tailored community outreach efforts may prove essential to achieve more equitable and optimal adult influenza vaccination coverage. The Healthy People 2030 goal of 30% nonreceipt remains ambitious given the lack of measurable progress over the past decade.^[3]^ Effecting meaningful vaccine coverage improvements will require departing from status quo efforts. Influenza vaccines are widely available at medical offices, pharmacies, and retail clinics, though uninsured individuals may face out-of-pocket costs for both vaccination and routine medical visits. Potential impactful strategies warranting consideration include expanding insurance coverage and reduced out-of-pocket costs to alleviate financial barriers; implementing public reporting and health care quality improvement initiatives prompting providers to assess vaccination status during all clinical encounters; launching multimodal community campaigns via digital platforms to increase vaccine awareness and acceptance among younger adults using messaging tailored to common concerns like side effects; deploying care coordinators and community health workers to facilitate meaningful conversations about vaccination benefits with vulnerable subgroups; and expanding alternate vaccination access through pharmacies and retail clinics to engage those lacking strong primary care connections.^[14,24–27]^ Embracing such innovative solutions while coordinating diverse stakeholders will be imperative to equitably translate the aspirational national influenza vaccination goals into measurable public health gains over the coming decade.

Several approaches could improve influenza vaccination coverage among the identified high-risk groups. Healthcare systems could implement communication strategies aligned with young adult preferences, such as automated reminders through digital platforms and social media campaigns. Vaccination opportunities can be expanded into convenient community locations to reach individuals without recent health care visits, including retail clinics, pharmacies, and mobile vaccination sites. Development and communication of clear, accessible educational materials about vaccine benefits and availability could enhance understanding and engagement across all literacy levels. Future research could evaluate the effectiveness of these approaches for each high-risk group since focusing on these populations may yield the greatest improvements in overall vaccination coverage.

This population-based study had several notable strengths, including the generalizability of the findings to the noninstitutionalized US adult population given the nationally representative sampling. In addition, the NHIS evaluated health characteristics across diverse sociodemographic groups that may not be accurately captured in smaller studies. We also determined the relative predictive importance of covariates for influenza vaccine nonreceipt, enabling practical interpretation of the many statistically significant logistic regression outputs.^[12]^ Finally, our study objectives aligned with those of Healthy People 2030, which highlights the societal relevance of these results. However, some limitations should be acknowledged. First, as an observational cross-sectional survey, we could not draw causal conclusions regarding the associations between participant characteristics and vaccine coverage. Second, influenza vaccination coverage estimates were derived from reports by survey respondents, not vaccination records, and may be influenced by recall bias. Third, the study did not account for all potential residual confounders that may influence decisions about influenza vaccination such as attitudinal factors, social perceptions, vaccine availability, and vaccine costs. Future studies could address these unmeasured factors through questionnaires using qualitative assessments of vaccination decision-making. Fourth, exclusion of institutionalized populations and those without fixed addresses, which represent ≈2% of the US adult population, may limit the applicability of study findings to these subgroups. Future studies could assess vaccination coverage in correctional facilities, long-term care institutions, and through outreach programs serving those without fixed addresses to understand patterns in these populations. Finally, the NHIS does not collect data on the reasons for vaccine nonreceipt. Thus, while younger adults, those without recent health care contact, and those with less education represent key targets for intervention, further qualitative research should explore the opinions and values influencing vaccine hesitancy in these subgroups. This would enable focused approaches addressing the specific concerns that shape their decisions to forgo vaccination.

## 5. Conclusion

Influenza vaccine coverage in the overall US adult population remains well below public health goals. Significant disparities persist among subgroups like younger adults, those without recent health care visits, and individuals with lower educational attainment. There is an urgent need for expanded outreach efforts targeting vulnerable populations to address disparities in vaccine uptake.

## Author contributions

**Conceptualization:** Larry E. Miller, Ruemon Bhattacharyya, Samuel J. Katz, Mehul Bhattacharyya.

**Formal analysis:** Larry E. Miller.

**Investigation:** Larry E. Miller, Ruemon Bhattacharyya, Samuel J. Katz, Mehul Bhattacharyya, Anna L. Miller.

**Methodology:** Larry E. Miller, Ruemon Bhattacharyya, Samuel J. Katz, Mehul Bhattacharyya, Anna L. Miller.

**Supervision:** Larry E. Miller.

**Writing – original draft:** Larry E Miller.

**Writing – review & editing:** Ruemon Bhattacharyya, Samuel J. Katz, Mehul Bhattacharyya, Anna L. Miller.

**Project administration:** Samuel J. Katz.

**Resources:** Anna L. Miller.
